# Supplementary material for: Changes in Polyphenolics during Storage of Products Prepared with Freeze-Dried Wild Blueberry Powder
Source: Foods. 2020 Apr 9;9(4):466. doi: 10.3390/foods9040466 (PMC7231037; doi:10.3390/foods9040466)
Supplement: Supplementary file 1 [file foods-09-00466-s001.pdf]

SUPPLEMENTARY FIGURES

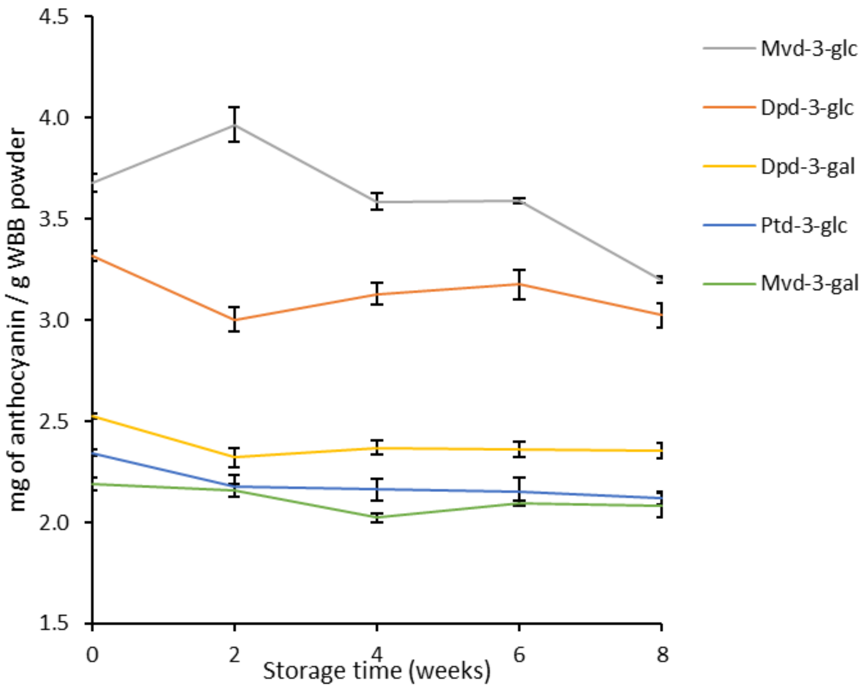

Figure S1. Five major individual anthocyanin content of blueberry ice pop stored at −20 °C over eight weeks. Bars represent standard error of the mean (n=3/time point). Anthocyanidins: Dpd = delphinidin, Mvd = malvidin, Ptd = petunidin. Sugar moiety: gal = galactose, glc = glucose.

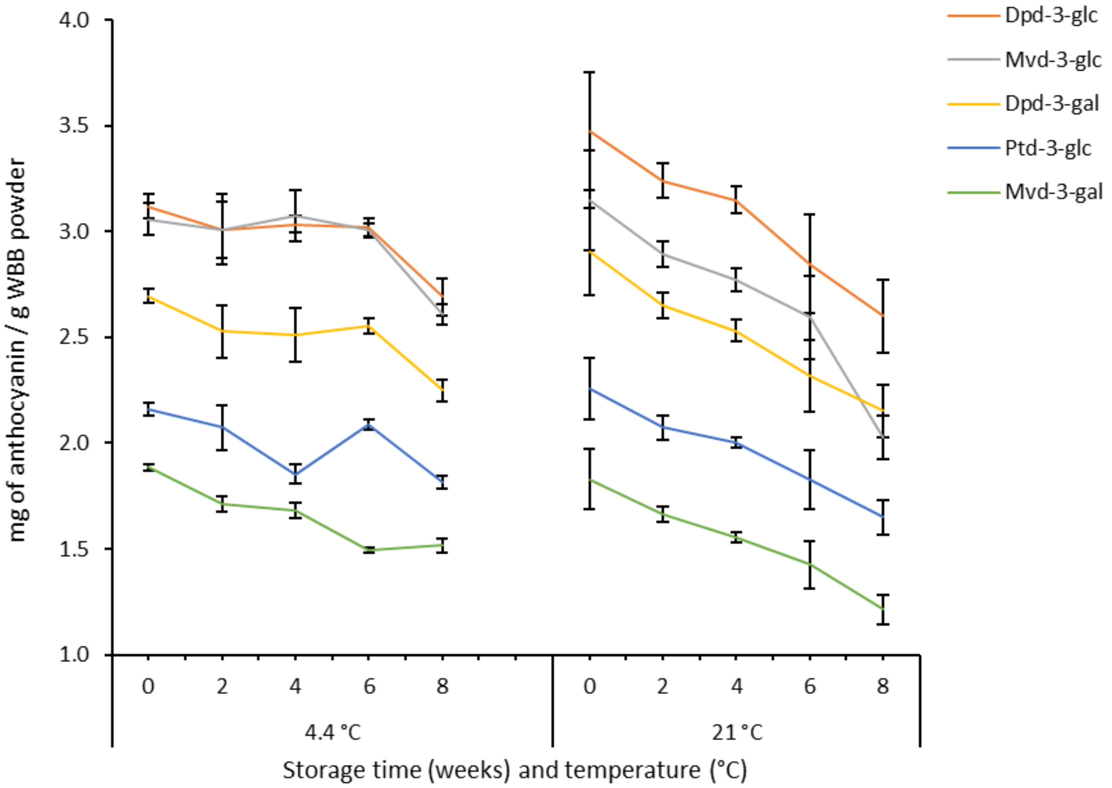

Figure S2. Five major individual anthocyanin content of blueberry oatmeal bar stored at 4.4 and 21 °C over eight weeks. Bars represent standard error of the mean (n=3/time point). Anthocyanidins: Dpd = delphinidin, Mvd = malvidin, Ptd = petunidin. Sugar moiety: gal = galactose, glc = glucose.

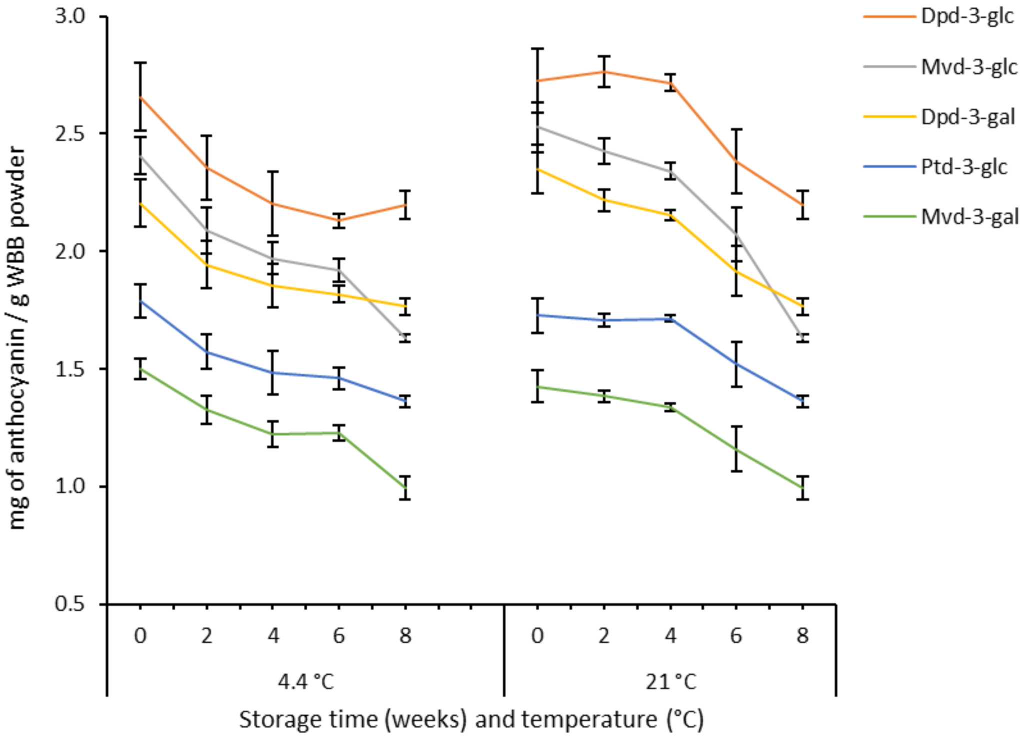

Figure S3. Five major individual anthocyanin content of blueberry graham cracker cookie stored at 4.4 and 21 °C over eight weeks. Bars represent standard error of the mean (n=3/time point). Anthocyanidins: Dpd = delphinidin, Mvd = malvidin, Ptd = petunidin. Sugar moiety: gal = galactose, glc = glucose.

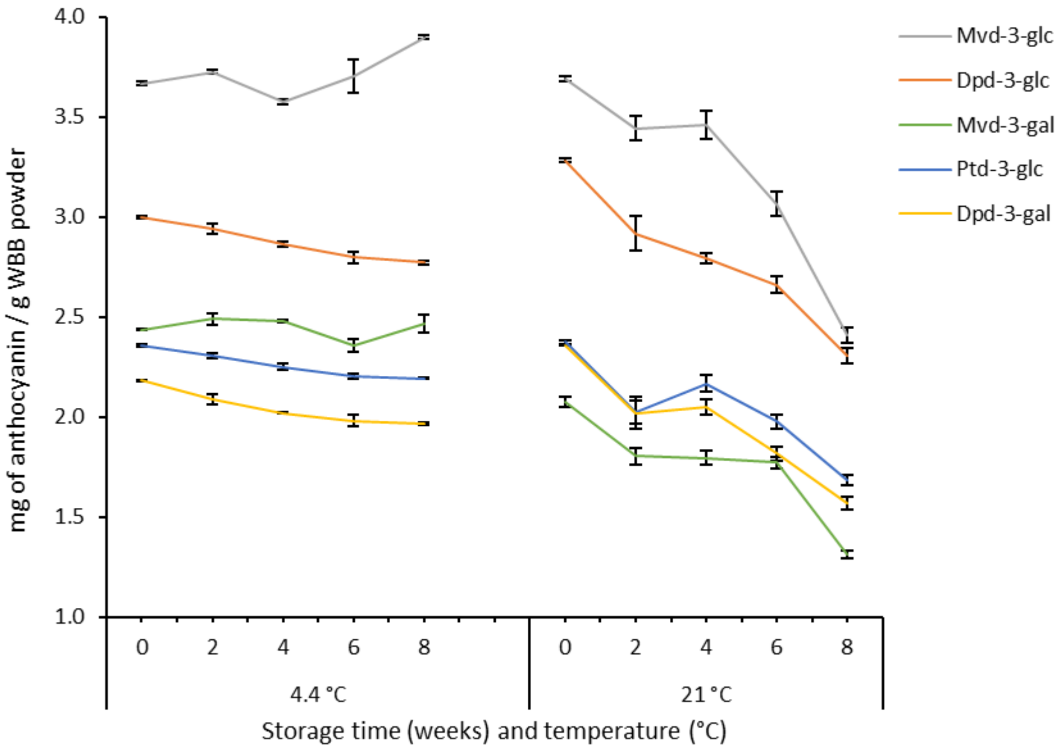

Figure S4. Five major individual anthocyanin content of blueberry juice stored at 4.4 and 21 °C over eight weeks. Bars represent standard error of the mean (n=3/time point). Anthocyanidins: Dpd = delphinidin, Mvd = malvidin, Ptd = petunidin. Sugar moiety: gal = galactose, glc = glucose.

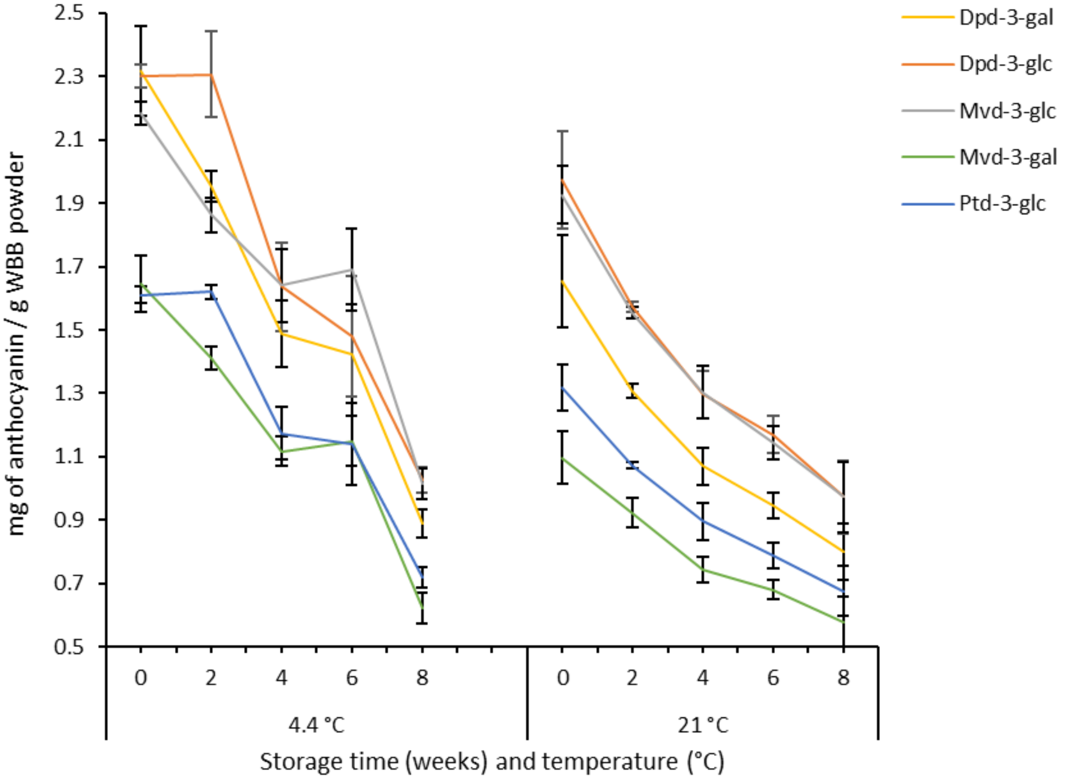

Figure S5. Five major individual anthocyanin content of blueberry gummy stored at 4.4 and 21 °C over eight weeks. Bars represent standard error of the mean (n=3/time point). Anthocyanidins: Dpd = delphinidin, Mvd = malvidin, Ptd = petunidin. Sugar moiety: gal = galactose, glc = glucose.
